# Dispositional Mindfulness and Subjective Time in Healthy Individuals

**DOI:** 10.3389/fpsyg.2016.00786

**Published:** 2016-05-31

**Authors:** Luisa Weiner, Marc Wittmann, Gilles Bertschy, Anne Giersch

**Affiliations:** ^1^INSERM U1114Strasbourg, France; ^2^Pôle de Psychiatrie et de Santé Mentale, Hôpitaux Universitaires de StrasbourgStrasbourg, France; ^3^Institute for Frontier Areas of Psychology and Mental HealthFreiburg, Germany

**Keywords:** time perception, dispositional mindfulness, duration estimation, passage of time, duration production

## Abstract

How a human observer perceives duration depends on the amount of events taking place during the timed interval, but also on psychological dimensions, such as emotional-wellbeing, mindfulness, impulsivity, and rumination. Here we aimed at exploring these influences on duration estimation and passage of time judgments. One hundred and seventeen healthy individuals filled out mindfulness (FFMQ), impulsivity (BIS-11), rumination (RRS), and depression (BDI-sf) questionnaires. Participants also conducted verbal estimation and production tasks in the multiple seconds range. During these timing tasks, subjects were asked to read digits aloud that were presented on a computer screen. Each condition of the timing tasks differed in terms of the interval between the presentation of the digits, i.e., either short (4-s) or long (16-s). Our findings suggest that long empty intervals (16-s) are associated with a relative underestimation of duration, and to a feeling that the time passes slowly, a seemingly paradoxical result. Also, regarding more mindful individuals, such a dissociation between duration estimation and passage of time judgments was found, but only when empty intervals were short (4-s). Relatively speaking, more mindful subjects showed an increased overestimation of durations, but felt that time passed more quickly. These results provide further evidence for the dissociation between duration estimation and the feeling of the passage of time. We discuss these results in terms of an alerting effect when empty intervals are short and events are more numerous, which could mediate the effect of dispositional mindfulness.

## Introduction

The subjective experience of duration is modulated by a number of contextual and individual factors independent of actual physical time. That is, the same time interval is experienced differently across individuals. Thus, time perception is involved in how one may feel according to the event taking place, and may play a crucial role in how one makes decisions in everyday life. For instance, if an individual overestimates the duration of a specific situation encountered in the past, during which time seemed to ‘drag,’ he might feel inclined to avoid it. Subjective time thereby involves at least two aspects – the judgment of the passage of time (i.e., how fast the time seems to pass) and the estimation of duration (i.e., how long an event seemed to have lasted), experiences which can vary independently of each other (Wearden et al., 2014). Personality traits, the nature of an event (as more or less attention capturing), and momentary physiological and psychological states are all involved in duration judgments. Here we designed a study in order to evaluate the relationship between dispositional mindfulness in a healthy population and different aspects of the experience of time in the multiple seconds range, such as the amount of events presented during the interval, and the kind of duration judgment required in the task – regarding the judgment of the passage of time and the estimation of duration.

Several factors should be controlled for when assessing empirically the experience of time. Findings suggest that the amount and the kind of events presented during an interval affect how alerting the event is judged to be, and thus how fast the passage of time and duration are estimated (Droit-Volet and Meck, 2007; Zakay, 2014). Essentially, the instructions given to participants before the actual task influence the judgment of time. When subjects are unaware that they are going to be asked questions about time (i.e., retrospective timing after the event or an interval has elapsed), they have to rely on memory retrieval. The more events and contextual changes are encoded and stored in memory the longer the duration is judged to be (Zakay and Block, 2004; Zakay, 2014). In contrast, when subjects are informed that the task they are going to perform is related to time (i.e., prospective timing), duration is estimated based on the attentional resources allocated to the task, i.e., duration estimation (rather than to the events). The more resources are allocated for timing, the longer the prospective duration judgment (Brown, 1997; Zakay and Block, 2004).

Multiple studies have found results consistent with this model (e.g., Block and Zakay, 1997; Wittmann and Paulus, 2008). Yet discordant results have also been reported in the literature, which seem to be related to the type, duration and complexity of information processed during the timed intervals (Wearden et al., 2014). Wearden (2008) included passage of time judgments in his study and found that, in a retrospective timing task, high information processing load (i.e., semantic vs. visual processing of words) was associated with a slowing of the passage of time when durations were relatively short (i.e., 18 s), and a speeding of the passage of time when durations were long (i.e., 72 s). However, there was no effect of the experimental manipulations on duration estimation. This dissociation between passage of time and duration judgments (see also Watt, 1991) suggests that passage of time judgments are particularly sensitive to the content of the events timed and how one subjectively reacts to this content (Wearden et al., 2014). All in all, these results first of all imply that the amount and the kind of events should be controlled for, and second, that the way subjects react to the amount of information during an interval has to be considered: the information may draw their attention or bore them depending on their disposition. Therefore, the experience of duration might be affected by subjective dimensions, such as mindfulness as a trait, and personality traits like impulsivity, or the tendency to ruminate.

Mindfulness affects the way individuals attend to the environment, regardless of the kind of events taking place. Mindfulness has been defined as intentionally focusing one’s attention on experiences occurring in the present moment in a non-judgmental way, i.e., attending to and accepting events as they occur (Kabat-Zinn, 1990). It is a multi-faceted concept that can be either conceptualized as a personality trait, or as a skill that can be trained. People who are more mindful are more able to allocate attention to each present moment, regardless of the information processing load. This would extend even to periods without specific events, during which more mindful subjects tend to attend to each moment in an accepting way. Besides effects on attention, mindfulness may also affect time perception (Wittmann and Schmidt, 2014). One of the main aspects that link mindfulness to time perception is its focus on moment-to-moment awareness. Several studies suggest that there is a relationship between mindfulness training and the experience of duration (Berkovich-Ohana et al., 2012; Kramer et al., 2013; Sucala and David, 2013; Droit-Volet et al., 2015; Wittmann et al., 2015) but there is only few data regarding the impact of mindfulness as a personality trait. In one study, Wittmann et al. (2014) examined the relationship between dispositional mindfulness, evaluated via two self-report questionnaires (FMI, Walach et al., 2006; CHIME, Bergomi et al., 2013) and the performance on prospective timing tasks. The authors found a relationship between elevated levels of self-rated mindfulness, particularly the ‘acceptance’ subscale of the FMI, and increased time discrimination accuracy in the milliseconds and seconds range. However, this study did not assess the judgment of the passage of time, and the impact of the nature of the task (e.g., with more or less events) on the experience of duration.

There are also other personality traits that might affect duration judgments. Conceptually, impulsivity is on the other end of a spectrum with trait mindfulness and self-control, and several studies (e.g., Peters et al., 2011; Wittmann et al., 2014, 2015) found an inverse relationship between dispositional mindfulness and impulsivity. Furthermore, like mindfulness, time perception seems to be related to impulsive behavior. For example, when making choices between smaller immediate versus larger delayed outcomes, impulsive individuals tend to prefer the smaller immediate outcome more often as compared to more self-controlled participants, who choose more often the larger delayed outcomes (e.g., Baumann and Odum, 2012). This choice bias in impulsive individuals has been found to be related to an overestimation of long durations (Wittmann and Paulus, 2008; Baumann and Odum, 2012). Moreover, in patients with psychiatric conditions characterized by an increased impulsivity, such as borderline personality disorder, substance-use disorder, and ADHD, an overestimation and under-production of intervals ranging from seconds to minutes has been found consistently (e.g., Barkley et al., 2001; Smith et al., 2002; Berlin and Rolls, 2004; Berlin et al., 2004; Wittmann et al., 2007). However, to our knowledge, these studies did not partial out the effect of facets of impulsivity on time perception. Several factor analytic studies suggest indeed that, like mindfulness, impulsivity is a multi-faceted concept (e.g., Stanford et al., 2009), including a (i) cognitive subtrait, involved in making cognitive decisions, a (ii) motor subtrait, involved in acting without thinking, and a (iii) non-planning subtrait, characterized as a present-orientation or a lack of “futuring” (Patton et al., 1995; Stanford et al., 2009). The non-planning subtrait seems to be particularly related to duration judgments, and can be expected to predict performance in timing tasks. On the other hand, the motor subtrait might be selectively involved in duration production. In addition, none of the previous studies with highly impulsive individuals included judgments of the passage of time nor did they partial out the specific contribution of different personality traits (mindfulness, impulsivity or rumination) on estimation of duration.

Here we aimed at taking into account the two personality traits of mindfulness and impulsivity which have been associated with the experience of time. In addition, we evaluated the contribution of the tendency to ruminate as well as current depressive symptoms, because they might represent confounding factors (Thönes and Oberfeld, 2015). Rumination has been defined as a repetitive and analytical pattern of thinking found to be a vulnerability trait for the development of depression (Aldao et al., 2010). According to the Response Styles Theory (RST; Nolen-Hoeksema, 1987), rumination includes both self-reflection, considered to be an adaptive form of rumination, and brooding, i.e., a repetitive focus on one’s negative emotions, considered to be maladaptive (Treynor et al., 2003). Maladaptive rumination is often accompanied by low mood, and a feeling that time is slowed down (e.g., Bschor et al., 2004; Boschloo et al., 2013; Marchetti et al., 2014). Conceptually, maladaptive rumination has been positively associated with impulsivity on the one hand, and negatively associated with mindfulness on the other hand (e.g., Paul et al., 2013; Marchetti et al., 2014; Selby et al., 2014; Crawley, 2015). Unlike brooding, self-reflection might be associated with mind-wandering and positive mood (Marchetti et al., 2014). Both aspects of rumination may thus have a distinct impact on time perception. This suggests that both facets associated with rumination tendency and current depressive mood should be taken into account when evaluating the specific contribution of mindfulness-proneness to duration judgments.

In our study, all subjects were tested in several experimental conditions, aimed at manipulating the amount of secondary-task events during duration judgments. The amount of events can have two opposite effects, as described above: it can divert attention from encoding duration, or it can lead subjects to feel less bored and be more attentive. To contrast these two possibilities, we manipulated the duration of empty intervals (4 vs. 16 s) between secondary-task events in a series of prospective tasks of duration estimation (32-s and 128-s duration) and duration production (30-s and 60-s duration). In addition to the duration judgments, we asked subjects to evaluate the subjective speed of the passage of time after the verbal estimation task. If subjects allocate more attention to time when empty intervals are long, then we should find a relative overestimation of durations for 16-s empty intervals. If the feeling of the passage of time is directly derived from duration estimation, an overestimation of duration should be associated with the feeling that time passes slowly. The feeling of the passage of time might be dissociated from duration estimation, however, (Wearden et al., 2014; Wearden, 2015). For example, long empty intervals may not affect attention allocation, but may be essentially boring. Reversely, short empty intervals may have an alerting effect. In that case, long empty intervals might lead subjects to evaluate the passage of time as being slower compared to shorter ones. Self-report questionnaires were used in order to evaluate the relationship between performance in these different conditions and personality traits. Our study design should be able to partial out the specific impact of mindfulness-proneness on duration evaluation and/or on the feeling of the passage of time, and to check whether such effects are independent or not from impulsivity, rumination tendency, and current mood.

## Materials and Methods

### Participants

For this cross-sectional study design, 117 healthy individuals aged 17–56 years (*M* = 26, *SD* = 7.7) were recruited among the staff and students at the University Hospital of Strasbourg. Women constituted 59% of the sample. Out of the 117 participants, 78 were university students, mostly in psychology and medical schools. It is to be noted that the results reported below were similar when only the university students were included in the analyses. Participants provided informed consent prior to inclusion in the study in accordance with the Declaration of Helsinki.

### Materials and Procedures

#### Timing Tasks

##### Equipment and stimuli

Time estimation and production tasks were generated via the Microsoft PowerPoint software (Wittmann et al., 2015). The presentation was displayed on a 17′ LCD color monitor. Participants viewed the display from a distance of 60 cm. During the timing tasks, digits were displayed, white on a black background, and were well-contrasted. Digits were 2.4° of visual angle high (1 cm corresponds to 1° of visual angle at a distance of 57 cm). Digits were presented on the center of the screen inside a red-lined square that measured 4.8° of visual angle and they were displayed for the duration of 1 s. The keyboard was used when motor responses were required. Four verbal estimation tasks, followed by a subjective judgment of the passage of time, were administered. Following the estimation tasks, four production tasks were administered.

##### Procedure for the verbal estimation and judgment of the passage of time tasks

Participants were asked to read aloud the numbers that were presented on the center of the computer screen, and estimate the duration of the task when it was over. The secondary number reading task was meant to prevent counting, and we additionally asked subjects to avoid counting while performing the task. Following the verbal estimation of the duration, subjects were asked to complete a visual analog scale (VAS), ranging from 1 (time passed very fast) to 10 (time passed very slowly), in order to evaluate their subjective perception of the passage of time during the estimation task. The conditions of the verbal estimation task differed in terms of their total duration (32 s vs. 128 s), as well as the stimulus onset asynchrony (SOA) between the digits that were presented and read aloud (mean = 4 s vs. mean = 16 s). The four conditions were presented in random order. In the 4-s SOA conditions, SOA actually ranged from 3 s to 5 s (mean = 4 s), and interval durations were presented in a random manner, in order to avoid anticipation. Likewise, in the 16-sec SOA conditions, SOA actually ranged from 13 to 19 s (mean = 16 s), and interval durations were presented in a random manner.

##### Procedure for the production tasks

Participants were asked to read aloud the numbers that were presented on the center of the screen, and press the “escape” button on the computer keyboard when 30 s (4 s vs. 16 s SOA between numbers) or 60 s (4 s vs. 16 s SOA between numbers) had passed. Like the estimation tasks, the secondary number reading task was meant to prevent counting, and we additionally asked subjects to avoid counting while performing the task. The actual time lapse produced by the participants was recorded via a timer. Like the estimation tasks, production tasks differed in terms of the duration expected (30 vs. 60 s), and the SOA between the digits that were presented (mean = 4 s vs. mean = 16 s). The four conditions were presented in a random order.

#### Self-Report Questionnaires

Participants filled-out four self-report questionnaires in-between verbal estimation and production task conditions. Questionnaires were administered in a fixed order.

##### Mindfulness

The Five Facets Mindfulness Questionnaire (FFMQ; Baer et al., 2008) is a 39-item self-report questionnaire which contains items rated on a five-point scale ranging from 1 (never or rarely true) to 5 (very often or always true). Mindfulness is measured on the basis of a five-dimensional structure with the factor ‘observing’ referring to the awareness of internal and external experiences; the factor ‘describing’ consisting of items related to ‘labeling internal experiences with words’; the ‘acting with awareness’ factor referring to the attention one pays to present activities; the ‘non-judgment of inner experiences’ factor relating to ‘the tendency to take a non-evaluative stance toward thoughts and feelings’; and the ‘non-reactivity to inner experience’ factor referring to the tendency to allow ‘thoughts and feelings to come and go.’ This questionnaire has good psychometric properties, and was constructed based on the items of five other mindfulness questionnaires. Indeed, Cronbach’s alpha for all of the five factors of the FFMQ are higher than 0.75, suggesting good internal consistency (Heeren et al., 2011). Because of its multifaceted nature, the FFMQ is supposed to reliably measure the complex construct of mindfulness (Sauer and Baer, 2010). The version of the scale we used has been validated in French (Heeren et al., 2011).

##### Impulsivity

The Barratt Impulsiveness Scale (BIS-11; Patton et al., 1995) is a 30-item self-report questionnaire with items rated on a 4-point scale ranging from 1 (rarely) to 4 (almost always). Items are grouped into three subscales corresponding to non-planning impulsivity (“I plan tasks carefully”), motor impulsivity (“I do things without thinking”), and cognitive impulsivity (“I concentrate easily”). Patton et al. (1995) reported an Alpha coefficient for the BIS total score of 0.82, suggesting an overall good internal consistency. The French version of the scale has been validated (Baylé et al., 2000).

##### Rumination

The Ruminative Response Scale (RRS; Nolen-Hoeksema and Morrow, 1991; Treynor et al., 2003) is a 22-item self-report questionnaire which evaluates two aspects of trait-rumination: ‘Brooding’ (five items) taps passive rumination on negative mood and is considered to be maladaptive whereas ‘reflection’ (five items) refers to active efforts to understand one’s negative feelings, and is considered to be adaptive. A total score is obtained *via* the sum of the 22 items. Items are rated on a scale of 1 (“almost never”) to 4 (“almost always”). In their psychometric analysis of the reflection and brooding subscales of the RRS, Treynor et al. (2003) found an Alpha coefficient of 0.72 and 0.77, respectively. The French version of the scale has been validated (Jermann et al., 2010).

##### Mood

The Beck Depression Inventory short-form (BDI-sf; Beck and Beamesderfer, 1974) is a 13-item self-report scale that measures the severity of depressive symptoms. Items are rated on a scale ranging from 0 (the symptom is absent) to 3 (maximum severity). This scale has been validated in French (Bourque and Beaudette, 1982).

## Results

Analyses were undertaken using the Statistica^®^ software. For each one of the three timing tasks – verbal estimation, judgment of the passage of time, and production –, we conducted a repeated measures ANOVA with two within-subject factors, task duration (32 vs. 128 s) and SOA between successive numbers (4 s vs. 16 s).

### Verbal Estimation, Judgment of the Passage of Time, and Temporal Production

**FIGURE 1:**
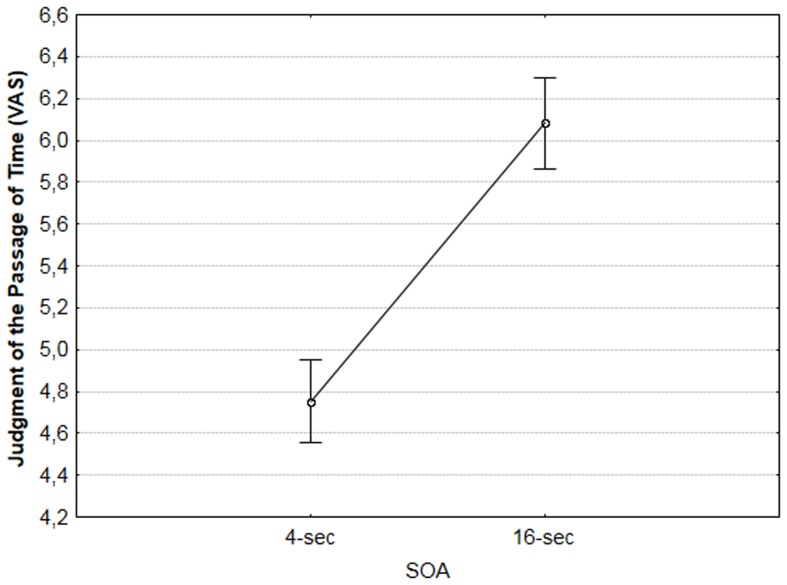
**Effect of the SOA between numbers on the judgment of the passage of time (VAS)**.

Overall, participants on average overestimated durations in both the 32-s and the 128-s duration conditions (mean = 39.41 s *SD* = 24.01 and mean = 144.23 s, *SD* = 78.08, respectively). In the verbal estimation tasks, there was no significant main effect of the SOA, *F*(1,116) = 3.06, *p* = 0.08, η^2^ = 0.026, but duration had a significant effect, *F*(1,116) = 333.48, *p* < 0.001, η^2^ = 0.742 (cf. Supplementary Material for means and standard errors across conditions in this task). As expected, estimations were longer when the actual durations were longer (128 s) as compared to when they were shorter (32 s). In the judgment of the flow of time, there was a significant main effect of both the SOA and duration, *F*(1,116) = 66.76, *p* < 0.001, η^2^ = 0.366, and *F*(1,116) = 161.33, *p* < 0.001, η^2^ = 0.582, respectively. Regardless of the duration condition, subjects judged the time to pass faster when the SOA was short (4 s), compared to when it was long (16 s; cf. **Figure 1** and Supplementary Material for means and standard errors across conditions in this task). Also, as expected, subjects judged the time to pass faster when the actual duration was short (32 sec), compared to when it was long (128 s).

**FIGURE 2:**
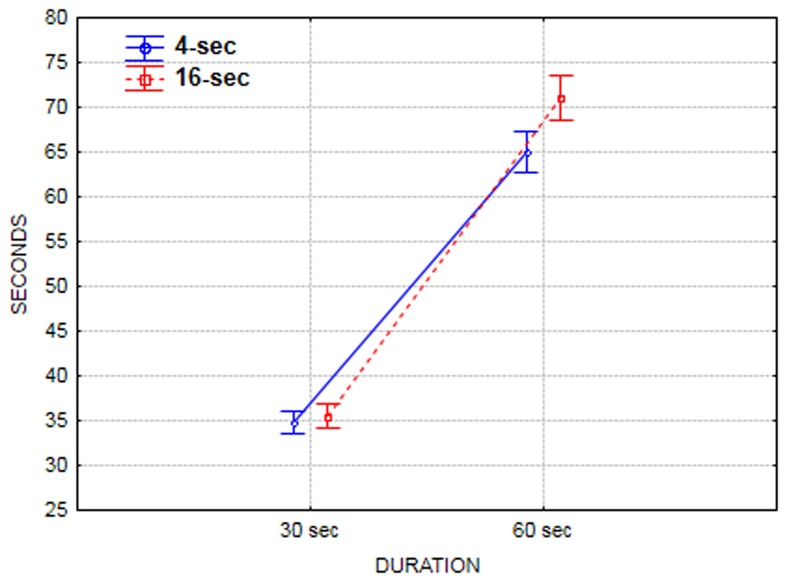
**Performance in the production task as function of the duration and the SOA**.

In the production tasks, on average, subjects overproduced durations in both the 30-s and the 60-s duration conditions (mean = 35.20, *SD* = 13.64, and mean = 67.85 and *SD* = 26.07, respectively). Moreover, there were significant main effects of both the SOA and the duration on the production performance, *F*(1,116) = 9.11, *p* < 0.001, η^2^ = 0.072, and *F*(1,116) = 466.35, *p* < 0.001,η^2^ = 0.800, respectively. As expected, subjects produced longer intervals when the duration was long (60 s), compared to when it was short (30 s). More interestingly, subjects overproduced more when the SOA was long (16 s) compared to when it was short (4 s), regardless of the task duration. There was a significant duration X SOA interaction, *F*(1,116) = 6.24, *p* = 0.01, η^2^ = 0.052. *Post hoc* Tukey test analyses revealed that only in the 60-s duration condition SOA had a significant effect on performance, with subjects overproducing more when the SOA was long (16 s) compared to when it was short (4 s; cf. **Figure 2** and Supplementary Material for means and standard errors across conditions in this task).

### Correlation Analyses between Psychological Dimensions

Regarding the correlation results reported below, in addition to presenting significance levels of *p* < 0.05 and *p* < 0.01, we applied a rigorous method of alpha level adjustment for multiple comparisons, the false discovery rate (FDR) method. FDR is a multiple comparisons correction, developed by Benjamini and Hochberg (1995), where we set the initial *p*-value to 0.05.

We conducted a first order correlation analyses in order to assess the relationship between the psychological dimensions evaluated via the questionnaires, namely trait-mindfulness, current mood, trait-impulsivity, and trait-rumination. Our aim was to check whether we could replicate the typically found relationships between mindfulness, impulsivity, rumination, and mood. Hence, we report here the correlations between different facets of mindfulness and different facets of these psychological dimensions. Overall, our results suggest that subjects who are more mindful, are also less impulsive (**Table 1**), ruminate less (**Table 2**), and report less depressive mood (**Table 3**). There is only one correlation going against those associations, i.e., a positive relationship between one subscale of the FFMQ, ‘observing’ and the subscales as well as the total score on the RRS. These correlations suggest that subjects, who observe more their inner experiences, are also more prone to rumination.

**Table 1:** Relationship between mindfulness and impulsivity.

	FFMQ Observing	FFMQ Describing	FFMQ Acting with awareness	FFMQ Non-reactivity	FFMQ Non-judgment	FFMQ Total
BIS non-planning	-0.01	-0.19^∗^	-0.27^∗∗∗^	-0.16	-0.12	-0.28^∗∗∗^
BIS Motor	0.04	0.16	-0.23^∗∗∗^	0.03	0.06	0.01
BIS Cognitive	0.08	-0.23^∗∗∗^	-0.51^∗∗∗^	0.02	-0.41^∗∗∗^	-0.42^∗∗∗^
BIS Total	0.05	-0.12	-0.45^∗∗∗^	-0.07	-0.19^∗^	-0.31^∗∗∗^


*^∗^*p* < 0.05; ^∗∗∗^FDR corrected FFMQ, Five Facets Mindfulness Questionnaire; BIS, Barratt Impulsiveness Scale.*

**Table 2:** Relationship between mindfulness and rumination.

	FFMQ Observing	FFMQ Describing	FFMQ Acting with awareness	FFMQ Non-reactivity	FFMQ Non-judgment	FFMQ Total
RRS brooding	0.21^∗∗∗^	-0.05	-0.25^∗∗∗^	-0.36^∗∗∗^	-0.41^∗∗∗^	-0.30^∗∗∗^
RRS reflection	0.37^∗∗∗^	0.14	-0.20^∗∗∗^	-0.09	-0.21^∗∗∗^	0.03
RRS total	0.34^∗∗∗^	-0.01	-0.40^∗∗∗^	-0.19^∗^	-0.38^∗∗∗^	-0.21^∗∗∗^


*^∗^*p* < 0.05; ^∗∗∗^FDR corrected; RRS, Ruminative Responses Scale; FFMQ, Five Facets Mindfulness Questionnaire.*

**Table 3:** Relationship between mindfulness and mood.

	FFMQ Observing	FFMQ Describing	FFMQ Acting with awareness	FFMQ Non-reactivity	FFMQ Non-judgment	FFMQ Total
BDI-sf	0.07	-0.21^∗^	-0.29^∗^^∗^^∗^	-0.14	-0.52^∗^^∗^^∗^	-0.40^∗^^∗^^∗^


*^∗^*p* < 0.05; ^∗∗∗^FDR corrected; FFMQ, Five Facets Mindfulness Questionnaire; BDI, Beck Depression Inventory – short form.*

### Regression Analyses

The regressions between the psychological dimensions suggest that several aspects of personality and mood are related to dispositional mindfulness. Any correlation between performance and the FFMQ may thus be partly due to another trait of personality. Thus, we conducted a multiple regression analysis to distinguish between the contribution of all evaluated aspects of personality and mood on timing performance. In the introduction, we suggested that specific facets of the personality dimensions assessed could be more specifically related to time perception than others. Because of this, all the personality subscales were entered into the model. Eleven predictors – all the subscales from four questionnaires, the FFMQ, the RRS, the BDI, and the BIS –, were simultaneously entered into the model: (i) 5 FFMQ subscores, (ii) 2 RRS subscores, (iii) BDI total score, and (iv) 3 BIS subscores.

#### Verbal Estimation

In the 4-s SOA conditions of the verbal estimation tasks, only the FFMQ subscales ‘acting with awareness’ and ‘observing’ were significant predictors of performance respectively in the 32-s (β = 0.25, *p* = 0.031) and 128-s (β = 0.25, *p* = 0.018) duration conditions (cf. **Table 4**; see the Supplementary Material for all data). Regardless of the duration condition, only when the SOA is relatively short (4-s) elevated levels of specific facets of mindfulness were associated with an overestimation of durations. In the 16-s SOA conditions of the verbal estimation tasks, there were no significant predictors of performance.

**Table 4:** Multiple linear regression analysis between verbal estimation (4-s SOA conditions) and significant psychological dimensions.

	Estimation 32-s (4-s SOA)^∗^				Estimation 128-s (4-s SOA)^∗∗^			
		
	*B*	*β*	*t*	*p*	*B*	*β*	*t*	*P*
FFMQ Observing	0.07	0.02	1.83	0.07	**3.24**	**0.25**	**2.40**	**0.02**
FFMQ acting with awareness	**1.00**	**0.25**	**2.19**	**0.03**	1.60	0.10	0.86	0.39


*B, regression coefficient; β, standardized regression coefficient; FFMQ, Five Facets Mindfulness Questionnaire. **^∗^**Δ*R*^*2*^ = 0.172, adjusted *R*^*2*^ = 0.085, *F*(11,105) = 1.981, *p* = 0.037. ^∗∗^Δ*R*^*2*^ = 0.142, adjusted *R*^*2*^ = 0.052, *F*(11,105) = 1.583, *p* = 0.114. Significant values in bold.*

#### Judgment of the Passage of Time

Significant predictors of performance in the judgment of the passage of time were only found in the 4-s SOA conditions. The ‘reflection’ subscale of the RRS was a significant predictor of performance in the 32-s condition (β = 0.24, *p* = 0.049). (cf. **Table 5**). An increased tendency to display an adaptive form of rumination was associated with the feeling that time passed slowly during the 32-s duration condition. On the other hand, the ‘acting with awareness’ subscale of the FFMQ was a significant predictor of performance in the 128-s duration conditions of this task (β = -0.25, *p* = 0.046) (cf. **Table 5**). Elevated levels of a specific facet of mindfulness (i.e., ‘acting with awareness’) were associated with an increased feeling that the time passed fast during the 128-s duration condition. In contrast, in the 16-s SOA conditions there were no significant predictors of performance.

**Table 5:** Multiple linear regression analysis between judgment of the passage of time (4-s SOA conditions) and significant psychological dimensions.

		Judgment of the passage of time 32-s (4-s SOA)^∗^				Judgment of the passage of time 128-s (4-s SOA)^∗∗^		
		
	*B*	*β*	*t*	*p*	*B*	*β*	*t*	*P*
FFMQ acting with awareness	-0.05	-0.17	-1.44	0.15	-**0.08**	-**0.247**	-**2.02**	**0.046**
RRS Reflection	**0.11**	**0.24**	**1.98**	**0.049**	0.03	0.06	0.51	0.61


*B, regression coefficient; β, standardized regression coefficient; FFMQ = Five Facets Mindfulness Questionnaire; RRS = Ruminative Responses Scale. **^∗^**Δ*R*^*2*^ = 0.088, adjusted *R*^*2*^ = -, *F*(11,105) = 0.917, *p* = 0.527. ^∗∗^Δ*R*^*2*^ = 0.051, adjusted *R*^*2*^ = -, *F*(11,105) = 0.514, *p* = 0.890. Significant values in bold.*

#### Production Tasks

In the 4-s SOA conditions of this task, only the FFMQ ‘describing’ subscale was a significant predictor of performance when subjects had to produce an interval of 30 s (β = -0.28, *p* = 0.011; cf. **Table 6**). Therefore, elevated levels of this specific facet of mindfulness were associated with a relative underproduction (i.e., overestimation) of duration. In the 16-s SOA conditions, only the RRS ‘reflection subscale’ was a significant predictor of performance in the 30-s condition of this task (β = 0.23, *p* = 0.048; cf. **Table 7**). An increased tendency to display this adaptive form of rumination was associated with a relative overproduction (i.e., underestimation) of duration.

**Table 6:** Multiple linear regression analysis between production tasks (4-s SOA conditions) and significant psychological dimensions.

	Production 30-s (4-s SOA)^∗^				Production 60-s (4-s SOA)^∗∗^			
		
	*B*	*β*	*t*	*p*	*B*	*β*	*t*	*P*
FFMQ Describing	**-0.68**	**-0.28**	**-2.58**	**0.01**	**-**0.45	**-**0.10	**-**0.87	0.38


*B, regression coefficient; β, standardized regression coefficient; FFMQ, Five Facets Mindfulness Questionnaire. **^∗^**Δ*R*^*2*^ = 0.156, adjusted *R*^*2*^ = 0.067, *F*(11,105) = 1.765, *p* = 0.0695. ^∗∗^Δ *R*^*2*^ = 0.059, adjusted *R*^*2*^ = –, *F*(11,105) = 0.599, *p* = 0.826. Significant values in bold.*

**Table 7:** Multiple linear regression analysis between production tasks (16-s SOA conditions) and significant psychological dimensions.

	Production 30-s (16-s SOA)^∗^				Production 60-s (16-s SOA)^∗∗^			
		
	*B*	*β*	*t*	*P*	*B*	*β*	*t*	*P*
RRS Reflection	**0.88**	**0.23**	**2.00**	**0.048**	**-**0.001	**-**0.00	**-**0.00	0.99


*B, regression coefficient; β, standardized regression coefficient; RRS, Ruminative Responses Scale. **^∗^**Δ*R*^*2*^ = 0.140, adjusted *R*^*2*^ = 0.050, *F*(11,105) = 1.554, *p* = 0.123. ^∗∗^Δ*R*^*2*^ = 0.107, adjusted *R*^*2*^ = 0.013, *F*(11,105) = 1.143, *p* = 0.336. Significant values in bold.*

## Discussion

The study results suggest that duration perception and the feeling of the passage of time are influenced both by external events and by personality traits. Overall, there was no effect of the length of the empty interval (4-s *vs*. 16-s) on verbal estimates of duration, but long empty intervals (16-s) led to a relative overproduction (i.e., underestimation) of durations. In addition, compared to short empty intervals (4-s), long empty intervals (16-s) were associated with a slowing of the feeling of the passage of time. Thus, we found a dissociation between passage of time and duration judgments: the feeling that time passes slowly is associated with estimations that durations are shorter than they are. Regarding personality dimensions, a dissociation between passage of time and duration judgments was also found: when empty intervals were short (4-s) increased levels of self-reported mindfulness were associated with both an overestimation of durations, and with a feeling that the time passes fast.

The results concerning the effects of different SOAs in the time perception tasks suggest that judgments of the passage of time were based on the feeling of boredom rather than on duration estimates *per se*. This assumption is crucial to interpret the whole pattern of results, and we will develop it first. Subjects knew in advance that they would have to estimate durations (prospective judgments), and we thus expected that more attention to duration itself would lead to lengthened duration estimates, i.e., overestimation and underproduction (Zakay and Block, 2004). Had this been the case, a high number of events, i.e., with 4-s SOAs, should have distracted subjects from time. In contrast, when empty intervals were long, attention should have been more easily diverted from the secondary ongoing task (reading numbers aloud) and be more focused on time itself, which should have led to an even stronger overestimation of durations. Hence durations should have been judged as being longer for long empty intervals (16-s SOAs) than for short empty intervals (4-s SOAs). This was not the case, however. Regarding duration estimation, there was no main effect of the SOA. Moreover, in the production tasks, long empty intervals led to an amplified overproduction, i.e., underestimation of durations. Hence, longer empty intervals of 16-s do not lead subjects to attend more to time, and, as a consequence, to lengthened duration estimates. In addition, the passage of time did not systematically co-vary with duration perception. There was only one case in which it did: Long duration conditions (128-s) were associated with a relative slowing of the passage of time compared to short ones (32-s). This suggests that subjects feel that time passes more slowly when actual durations are indeed longer. This could have been interpreted as a conflation of passage of time judgments with duration judgments. However, within the same duration conditions, subjects felt that the time passed faster when empty intervals were short (4-s) compared to when they were long (16-s), although duration estimates did not differ between the two conditions. If passage of time judgments had reflected duration perception *per se*, then a shortening of duration estimates should have been associated with a feeling that time passes fast (and conversely). Long empty intervals led to an overproduction, which, according to the standard cognitive pacemaker-accumulator model, corresponds to a shortening of duration estimates (Zakay and Block, 2004; Droit-Volet and Meck, 2007; Wearden et al., 2014). This relative shortening of duration should thus have been associated with a feeling that time passes faster. However, the results showed the reverse. Long empty intervals led to an overproduction (underestimation) of durations, but also to a feeling that time passes slowly. Similar dissociations between the subjective passage of time and duration estimation judgments were observed when considering psychological dimensions. Compared to other psychological dimensions, mindfulness was the main predictor of performance on duration estimation and the passage of time judgments for short (4-s SOA) empty intervals. However, high levels of mindfulness were associated with both a greater overestimation of duration, and with a faster passage of time. That is, we show a dissociation between duration estimation and the judgment of passage of time, as more mindful individuals in one condition relatively overestimated duration but also felt that the time passed faster. Again, if the evaluation of the passage of time had been directly related to duration estimation, overestimation of durations should have led to the feeling that time passed slowly, whereas we observed the reverse relation. This means that the judgment of the subjective passage of time reflects something different than duration estimation. Our results do not seem to be easily explained by boredom either.

Past studies have reported a relationship between boredom and a slowing of the passage of time (Watt, 1991; Wittmann and Paulus, 2008; Wearden et al., 2014). As suggested by Zakay (2014), boredom may not only induce the feeling that time passes slowly, but may also increase the attentional resources allocated to time. Indeed, in a boring situation, subjects would look forward to the end of the boring period, and thus would attend more to time, leading to a lengthening of duration estimation. If the 16-s empty interval is experienced as unusually long, it should lead subjects to both an underproduction, i.e., a lengthening of duration perception, and the feeling that time passes more slowly. As underlined above, this was not observed. Even if long (16-s) intervals were associated with a slowing of the passage of time, they did not lead subjects to attend more to time and, as a consequence, to an overestimation of durations. Wearden (2015) suggested recently that the feeling of the passage of time and duration estimation are two independent dimensions, and our results are consistent with this proposal. The duration of the tasks as a whole was rather short (i.e., up to 128-s for verbal estimation and 60-s for production tasks), and subjects may not have felt bored enough to attend more to time when empty intervals were long (16-s) compared to when they were short (4-s). However, the structure of our experiment may suggest yet another possibility. According to the model of Zakay and Block (2004), attention is allocated to either time or the task at hand, but not to both. This is particularly well adapted to everyday life, when a stimulating task possibly captures all one’s attention, leading subjects to ‘lose track of time.’ However, in the case of empty intervals, it is extremely difficult to control the kind of internal information processing that subjects might engage themselves in Zakay (1993). In non-stimulating situations, they could either attend to time (e.g., because they are bored), or mind-wander. It is perfectly possible that the regular interruption of empty intervals by the presentation of numbers prevented subjects from mind-wandering. This would not have been the case for 16-s intervals though, because they were exceptionally long. Hence, if mind-wandering was more frequent during long empty intervals, it might have paradoxically distracted subjects from attending to time, contrary to our initial predictions. If, at all, 4-s intervals may have produced a non-specific alerting effect, which might have helped subjects to be more attentive both to temporal and non-temporal events, thus leading to less overproduction when empty intervals were short (4-sec SOAs). An explanation in terms of alertness is consistent with results reported by Treisman (1963) and Wearden (2008), and recent studies by Wearden et al. (2014) and Droit-Volet and Wearden (2015). These authors suggest that increased arousal, which might also be translated as increased alertness, may be associated to both a lengthening of durations, and to a faster passage of time, as observed in the present study. Such a non-specific attentional effect might also explain the results regarding dispositional mindfulness.

To our knowledge, this is the first study to partial out the specific contributions of dispositional mindfulness on judgments of multiple-seconds intervals. Before developing this point, it is important to note that our evaluation of personality traits is consistent with the literature. We found a significant negative relationship between (i) dispositional mindfulness and impulsivity (e.g., Wittmann et al., 2014), (ii) mindfulness and rumination (e.g., Paul et al., 2013; Marchetti et al., 2014; Crawley, 2015), and (iii) mindfulness and current depressive symptoms (e.g., Shorey et al., 2014). The replication of results found in earlier studies suggests that self-report measures are reliable, and that these psychological dimensions are strongly related to each other. However, when all of them were taken into account, only dispositional mindfulness and reflection-rumination significantly predicted performance on both duration estimation and passage of time judgments. Indeed, in our study, mindfulness was associated with a greater overestimation of duration and a faster passage of time, and the opposite was found for reflection-rumination. Regarding passage of time judgments, as mentioned before, long empty intervals led to a feeling that time passed more slowly, whereas there was no effect of the length of the empty interval on verbal estimates of duration. When personality dimensions were taken into account, only when empty intervals were short (4-s SOA) did mindfulness and trait reflection-rumination predict performance on the feeling of the passage of time. This suggests that, regardless of personality traits, when empty intervals are long (16-s SOA), subjects as a whole feel that time passes more slowly. This might be akin to a ceiling effect, where 16-s intervals are too long to distinguish more mindful from less mindful individuals. In contrast, when empty intervals are short, personality traits such as mindfulness and trait reflection-rumination predict, in a distinctive manner, the way subjects feel that the time has passed – either fast or slow, respectively.

Since high levels of mindfulness were related to a relative speeding of the passage of time, this suggests that highly mindful subjects were rather less bored and more attentive to the secondary ongoing task, whereas the opposite would hold true for individuals with higher values in trait reflection-rumination, who were probably more bored and reported a slowing of the passage of time. The fact that more mindful subjects are more attentive to the ongoing task is consistent with previous studies showing improved performance on attention tasks in these subjects (e.g., Ainsworth et al., 2013). Also, consistent with this interpretation, it is interesting to note that ‘acting with awareness’ was the only mindfulness subscale which predicted performance on passage of time judgments in our study. This indicates that those individuals, who are more able to allocate attention to present non-temporal events, are those who feel that the time passes faster. In addition, being more mindful during the time perception task itself leads to longer duration estimates, as found in our study. As suggested above, this might be explained by a non-specific effect of attention. If mindful subjects are particularly attentive to present events, and if these events have a non-specific arousal effect, then these attention mechanisms would explain why highly mindful subjects overestimate durations, on the one hand, and feel that time passes faster, on the other hand. These results nevertheless should be interpreted with caution, since a high number of psychological dimensions were entered in our regression model, and only a few facets of mindfulness and rumination seemed to be related to time perception. Although we checked for multiple comparisons, it is difficult to exclude the possibility of false positives, and it is also possible that some relationships were overlooked due to the alpha adjustment for multiple testing.

There are a few limitations to this study. First of all, the majority of our sample comprised young students, which raises the question of the generalizability of our findings. Future studies should include more individuals in other age groups, since time perception abilities may change during the life-span (Ulbrich et al., 2007; Wearden, 2015). Second, the concept of mindfulness is derived from eastern Buddhist tradition, and whether self-report questionnaires are able to evaluate the psychological mechanisms involved in this concept is still a matter of debate (Grossman, 2011). Third, the design of our study makes it difficult to determine how individuals responded to empty intervals, and whether they felt bored during the task. Thus, boredom – as a personality trait and as a state related to the task – should be assessed directly in future studies. In addition, the mechanisms involved in passage of time judgments are still hypothetical, and deserve to be explored in a more thorough manner. Finally, the relationship between time perception and other psychological dimensions, such as the tendency to self-reflect or mind-wander, should be addressed in a more in-depth manner in future studies.

All in all, our results are nonetheless in line with findings reported by Wittmann et al. (2014) who suggested that, as a personality trait, mindfulness is associated with duration estimation. They are also consistent with results in experienced mindfulness meditators, since several studies have shown that meditators relatively overestimate durations in the milliseconds to seconds and also minutes range (Kramer et al., 2013; Sucala and David, 2013; Droit-Volet et al., 2015). Moreover, our findings add to those showing dissociation between duration judgments and the feeling of the passage of time (Wearden et al., 2014; Wearden, 2015).

## Author Contributions

AG and MW devised the study, with the help of LW. LW recruited and tested the subjects, and analyzed the results with the help of AG and MW. All authors (LW, MW, GB, and AG) interpreted the data. LW wrote the first draft of the manuscript. All authors (LW, MW, GB, and AG) reviewed and approved the manuscript.

## Conflict of Interest Statement

The authors declare that the research was conducted in the absence of any commercial or financial relationships that could be construed as a potential conflict of interest.
