# Supplementary material for: Dispositional Mindfulness and Subjective Time in Healthy Individuals
Source: Front Psychol. 2016 May 31;7:786. doi: 10.3389/fpsyg.2016.00786 (PMC4885856; doi:10.3389/fpsyg.2016.00786)
Supplement: Supplementary file 1 [file Table_1.DOC]

|  | **Estimation** | | **Passage of time** | | **Production** | |
| --- | --- | --- | --- | --- | --- | --- |
| SOA\Duration | **32-sec** | **128-sec** | 1. **32-sec** | **128-sec** | **30-sec** | **60-sec** |
| **4-sec** | 41.69 (2.11) | 146.91 (7.30) | 3.79 (0.15) | 5.71 (0.18) | 34.90 (1.24) | 64.98 (2.35) |
| **16-sec** | 37.12 (2.31) | 141.56 (7.15) | 5.11 (0.21) | 7.05 (0.16) | 35.50 (1.28) 71.01 (2.45) | 71.01 (2.45) |

Table 1: Means and standard errors across conditions of the timing tasks
