# Supplementary material for: Dispositional Mindfulness and Subjective Time in Healthy Individuals
Source: Front Psychol. 2016 May 31;7:786. doi: 10.3389/fpsyg.2016.00786 (PMC4885856; doi:10.3389/fpsyg.2016.00786)
Supplement: Supplementary file 2 [file Table_2.DOC]

**Table 2:** Multiple linear regression analysis between verbal estimation (4-sec SOA conditions) and psychological dimensions

|  | **Estimation 32-sec (4-sec SOA)*** | | | |  | **Estimation 128-sec (4-sec SOA)**** | | | |
| --- | --- | --- | --- | --- | --- | --- | --- | --- | --- |
|  | B | β | t | p |  | B | β | t | p |
| **FFMQ Observing** | .07 | .02 | 1.84 | 0.07 |  | **3.24** | **.25** | **2.40** | **.02** |
| **FFMQ Describing** | .67 | .16 | .15 | .13 |  | 1.52 | .11 | .98 | .33 |
| **FFMQ**  **acting with awareness** | **1.00** | **.25** | **2.19** | **.03** |  | 1.60 | .10 | .86 | **.**39 |
| **FFMQ non judgment** | -.07 | -.18 | - 1.46 | .15 |  | -1.75 | -.13 | -1.05 | .30 |
| **FFMQ non reactivity** | -.66 | -.14 | -1.44 | .15 |  | -.72 | -.04 | -.45 | .65 |
| **BIS Non planning** | -.20 | -.03 | -.34 | .74 |  | -2.06 | -.10 | -1.01 | .31 |
| **BIS Motor** | -.60 | -.12 | -1.13 | .26 |  | -1.65 | -.09 | -.88 | .38 |
| **BIS Cognitive** | .86 | .13 | 1.13 | .26 |  | 2.97 | .13 | 1.12 | .27 |
| **RRS Brooding** | -1.14 | -.16 | -1.30 | .20 |  | -4.03 | -.16 | -1.30 | .19 |
| **RRS Reflection** | -.01 | -.01 | -.02 | .98 |  | -1.90 | -.09 | -.75 | .45 |
| **BDI** | .51 | .08 | .72 | .47 |  | -.92 | -.04 | -.37 | .71 |

B, regression coefficient ; β, standardized regression coefficient ; FFMQ = Five Facets Mindfulness Questionnaire; BIS = Barratt Impulsiveness Scale; RRS = Ruminative Responses Scale; BDI = Beck Depression Inventory

*****Δ R^2^ = .172, adjusted R^2^ = .085, F (11,105) = 1.981, p =.037

** Δ R^2^ = .142, adjusted R^2^ = .052, F (11,105) = 1.583, p = .114
