# Supplementary material for: Dispositional Mindfulness and Subjective Time in Healthy Individuals
Source: Front Psychol. 2016 May 31;7:786. doi: 10.3389/fpsyg.2016.00786 (PMC4885856; doi:10.3389/fpsyg.2016.00786)
Supplement: Supplementary file 3 [file Table_3.DOC]

**Table 3: Multiple linear regression analysis between verbal estimation (16-sec SOA conditions) and psychological dimensions**

|  | **Estimation 32-sec (16-sec SOA)*** | | | |  | **Estimation 128-sec (16-sec SOA)**** | | | |
| --- | --- | --- | --- | --- | --- | --- | --- | --- | --- |
|  | B | β | t | p |  | B | β | t | p |
| **FFMQ Observing** | .05 | .01 | 1.06 | .29 |  | 2.17 | .17 | 1.60 | .11 |
| **FFMQ Describing** | .91 | .20 | 1.82 | .07 |  | 2.83 | .20 | 1.81 | .07 |
| **FFMQ**  **acting with awareness** | .16 | .04 | .31 | .75 |  | 1.37 | .10 | .85 | **.**40 |
| **FFMQ non judgment** | -.55 | -.13 | - 1.03 | .30 |  | -.07 | -.01 | -.04 | .96 |
| **FFMQ non reactivity** | -.66 | -.13 | -1.29 | .20 |  | -.67 | -.04 | -.42 | .67 |
| **BIS Non planning** | -.20 | -.03 | -.31 | .75 |  | -.70 | -.04 | -.34 | .73 |
| **BIS Motor** | -.06 | -.01 | -.10 | .92 |  | -2.16 | -.13 | -1.15 | .25 |
| **BIS Cognitive** | .13 | .02 | .16 | .87 |  | 3.09 | .14 | 1.15 | .25 |
| **RRS Brooding** | -1.18 | -.15 | -1.19 | .23 |  | .06 | .00 | .02 | .98 |
| **RRS Reflection** | -1.23 | -.18 | -1.52 | .13 |  | -3.56 | -.17 | -1.41 | .16 |
| **BDI** | 1.28 | .19 | 1.60 | .11 |  | 2.15 | .10 | .86 | .39 |

B, regression coefficient ; β, standardized regression coefficient ; FFMQ = Five Facets Mindfulness Questionnaire; BIS = Barratt Impulsiveness Scale; RRS = Ruminative Responses Scale; BDI = Beck Depression Inventory

*****Δ R^2^ = .114, adjusted R^2^ = .022, F (11,105) = 1.232, p =.275

** Δ R^2^ = .096, adjusted R^2^ = .001, F (11,105) = 1.009, p = .443
