# Supplementary material for: Dispositional Mindfulness and Subjective Time in Healthy Individuals
Source: Front Psychol. 2016 May 31;7:786. doi: 10.3389/fpsyg.2016.00786 (PMC4885856; doi:10.3389/fpsyg.2016.00786)
Supplement: Supplementary file 4 [file Table_4.DOC]

**Table 4:** Multiple linear regression analysis between judgment of the flow of time (4-sec SOA conditions) and psychological dimensions

|  | **Judgment of the flow of time 32-sec (4-sec SOA)*** | | | |  | **Judgment of the flow of time 128-sec (4-sec SOA)**** | | | |
| --- | --- | --- | --- | --- | --- | --- | --- | --- | --- |
|  | B | β | t | p |  | B | β | t | p |
| **FFMQ Observing** | -.02 | -.08 | -.76 | .45 |  | -.01 | -.04 | - .36 | .72 |
| **FFMQ Describing** | .02 | .08 | .71 | .48 |  | .00 | .01 | .13 | .89 |
| **FFMQ**  **acting with awareness** | -.05 | -0.17 | -1.44 | .152 |  | **- .08** | **-.25** | **-2.02** | **.04** |
| **FFMQ non judgment** | .01 | .03 | .025 | .80 |  | .04 | .12 | .86 | .39 |
| **FFMQ non reactivity** | .01 | .02 | .18 | .86 |  | -.01 | -.03 | -.25 | .80 |
| **BIS Non planning** | -.03 | -.08 | -.73 | .47 |  | .02 | .04 | .36 | .72 |
| **BIS Motor** | -.02 | -.05 | -.47 | .64 |  | -.02 | -.05 | -.45 | .65 |
| **BIS Cognitive** | .01 | .03 | .25 | .80 |  | -.04 | -.07 | -.60 | .55 |
| **RRS Brooding** | -.03 | -.05 | -.41 | .68 |  | -.05 | -.09 | -.66 | .51 |
| **RRS Reflection** | **.11** | **.24** | **1.98** | **.04** |  | .03 | .06 | .51 | .61 |
| **BDI** | .02 | .05 | .45 | .65 |  | .03 | .05 | .41 | .68 |

B, regression coefficient ; β, standardized regression coefficient ; FFMQ = Five Facets Mindfulness Questionnaire; BIS = Barratt Impulsiveness Scale; RRS = Ruminative Responses Scale; BDI = Beck Depression Inventory

*****Δ R^2^ = .088, adjusted R^2^ = -, F (11,105) = .917, p =.527

** Δ R^2^ = .051, adjusted R^2^ = --, F (11,105) =.514, p = .890
