# Supplementary material for: Dispositional Mindfulness and Subjective Time in Healthy Individuals
Source: Front Psychol. 2016 May 31;7:786. doi: 10.3389/fpsyg.2016.00786 (PMC4885856; doi:10.3389/fpsyg.2016.00786)
Supplement: Supplementary file 5 [file Table_5.DOC]

**Table 5:** Multiple linear regression analysis between judgment of the flow of time (16-sec SOA conditions) and psychological dimensions

|  | **Judgment of the flow of time 32-sec (16-sec SOA)*** | | | |  | **Judgment of the flow of time 128-sec (16-sec SOA)**** | | | |
| --- | --- | --- | --- | --- | --- | --- | --- | --- | --- |
|  | B | β | t | p |  | B | β | t | p |
| **FFMQ Observing** | .04 | .10 | .92 | .36 |  | .01 | .05 | .47 | .64 |
| **FFMQ Describing** | .01 | .03 | .23 | .82 |  | .02 | .07 | .60 | .55 |
| **FFMQ**  **acting with awareness** | -.01 | -.04 | -.30 | .76 |  | - .02 | -.08 | -.68 | .49 |
| **FFMQ non judgment** | -.01 | -.02 | -.13 | .90 |  | .01 | .02 | .16 | .87 |
| **FFMQ non reactivity** | -.00 | -.00 | -.00 | .99 |  | -.07 | -.20 | -1.94 | .05 |
| **BIS Non planning** | -.01 | -.02 | -.23 | .82 |  | .02 | .04 | .40 | .69 |
| **BIS Motor** | .00 | .01 | .10 | .92 |  | -.02 | -.045 | -.44 | .66 |
| **BIS Cognitive** | .07 | .10 | .81 | .42 |  | .02 | .05 | .37 | .71 |
| **RRS Brooding** | -.01 | -.02 | -.16 | .87 |  | .02 | .05 | .345 | .73 |
| **RRS Reflection** | -.00 | -.01 | -.05 | .96 |  | -.06 | -.13 | -1.12 | .27 |
| **BDI** | -.01 | -.01 | -.10 | .92 |  | .04 | .10 | .82 | .41 |

B, regression coefficient ; β, standardized regression coefficient ; FFMQ = Five Facets Mindfulness Questionnaire; BIS = Barratt Impulsiveness Scale; RRS = Ruminative Responses Scale; BDI = Beck Depression Inventory

*****Δ R^2^ = .029, adjusted R^2^ = -, F (11,105) = .284, p =.988

** Δ R^2^ = .081, adjusted R^2^ = --, F (11,105) =.843, p = .597
