# Supplementary material for: Dispositional Mindfulness and Subjective Time in Healthy Individuals
Source: Front Psychol. 2016 May 31;7:786. doi: 10.3389/fpsyg.2016.00786 (PMC4885856; doi:10.3389/fpsyg.2016.00786)
Supplement: Supplementary file 6 [file Table_6.DOC]

**Table 6:** Multiple linear regression analysis between production tasks (4-sec SOA conditions) and psychological dimensions

|  | **Production 30-sec (4-sec SOA)*** | | | |  | **Production 60-sec (4-sec SOA)**** | | | |
| --- | --- | --- | --- | --- | --- | --- | --- | --- | --- |
|  | B | β | t | p |  | B | β | t | p |
| **FFMQ Observing** | -.22 | -.01 | -.97 | .34 |  | -.58 | -.14 | -1.27 | .21 |
| **FFMQ Describing** | **- .68** | **-.28** | **-2.58** | **.01** |  | -.45 | -.10 | -.87 | .39 |
| **FFMQ**  **acting with awareness** | .04 | .02 | .14 | .89 |  | - .08 | -.02 | -.16 | .88 |
| **FFMQ non judgment** | .46 | .21 | 1.62 | .11 |  | .18 | .04 | .32 | .75 |
| **FFMQ non reactivity** | -.26 | -.10 | -.96 | .34 |  | .39 | .08 | .72 | .47 |
| **BIS Non planning** | -.57 | -.17 | -1.66 | .10 |  | -.38 | -.06 | -.55 | .58 |
| **BIS Motor** | .22 | .07 | .69 | .49 |  | .13 | .02 | .21 | .84 |
| **BIS Cognitive** | .68 | .18 | 1.51 | .13 |  | .68 | .09 | .76 | .45 |
| **RRS Brooding** | .56 | .14 | 1.08 | .28 |  | .95 | .12 | .91 | .37 |
| **RRS Reflection** | .00 | .00 | .01 | .99 |  | .25 | .04 | .29 | .77 |
| **BDI** | -.14 | -.04 | -.32 | .75 |  | -.98 | -.14 | -1.17 | .24 |

B, regression coefficient ; β, standardized regression coefficient ; FFMQ = Five Facets Mindfulness Questionnaire; BIS = Barratt Impulsiveness Scale; RRS = Ruminative Responses Scale; BDI = Beck Depression Inventory

*****Δ R^2^ = .156, adjusted R^2^ = .067, F (11,105) = 1.765, p =.0695

** Δ R^2^ = .059, adjusted R^2^ = --, F (11,105) =.599, p = .826
