# Supplementary material for: Dispositional Mindfulness and Subjective Time in Healthy Individuals
Source: Front Psychol. 2016 May 31;7:786. doi: 10.3389/fpsyg.2016.00786 (PMC4885856; doi:10.3389/fpsyg.2016.00786)
Supplement: Supplementary file 7 [file Table_7.DOC]

**Table 7:** Multiple linear regression analysis between production tasks (16-sec SOA conditions) and psychological dimensions

|  | **Production 30-sec (16-sec SOA)*** | | | |  | **Production 60-sec (16-sec SOA)**** | | | |
| --- | --- | --- | --- | --- | --- | --- | --- | --- | --- |
|  | B | β | t | p |  | B | β | t | p |
| **FFMQ Observing** | -.29 | -.13 | -1.24 | .22 |  | -.06 | -.01 | -.13 | .90 |
| **FFMQ Describing** | - .31 | -.13 | -1.15 | .25 |  | -.22 | -.05 | -.42 | .68 |
| **FFMQ**  **acting with awareness** | .07 | .03 | . 25 | .80 |  | - .47 | -.10 | -.87 | .39 |
| **FFMQ non judgment** | .40 | .18 | 1.38 | .17 |  | .33 | .07 | .57 | .57 |
| **FFMQ non reactivity** | .24 | .09 | .87 | .38 |  | .35 | .09 | .87 | .39 |
| **BIS Non planning** | -.37 | -.10 | -1.02 | .31 |  | -.51 | -.08 | -.73 | .46 |
| **BIS Motor** | .05 | .02 | .16 | .87 |  | 1.14 | .19 | 1.78 | .08 |
| **BIS Cognitive** | .91 | .24 | 1.956 | .053 |  | 1.00 | .14 | 1.11 | .27 |
| **RRS Brooding** | .27 | .06 | .49 | .62 |  | .99 | .12 | .94 | .35 |
| **RRS Reflection** | **.88** | **.23** | **2.00** | **.04** |  | -.00 | -.00 | -.00 | .99 |
| **BDI** | -.21 | -.06 | -.49 | .62 |  | -.90 | -.12 | -1.05 | .29 |

B, regression coefficient ; β, standardized regression coefficient ; FFMQ = Five Facets Mindfulness Questionnaire; BIS = Barratt Impulsiveness Scale; RRS = Ruminative Responses Scale; BDI = Beck Depression Inventory

*****Δ R^2^ = .140, adjusted R^2^ = .050, F (11,105) = 1.554, p =.123

** Δ R^2^ = .107, adjusted R^2^ = .013, F (11,105) = 1.143, p = .336
